# The importance of FDG PET/CT in the diagnostic process of the middle aortic syndrome in a 15-year-old boy patient with suspected systemic vasculitis and final diagnosis of Williams–Beuren syndrome

**DOI:** 10.1007/s00296-020-04550-3

**Published:** 2020-04-01

**Authors:** Violetta Opoka-Winiarska, Maria Barbara Tomaszek, Aleksandra Sobiesiak, Aleksandra Rybkowska, Monika Lejman, lIona Jaszczuk, Magdalena Maria Woźniak, Edyta Zielonka-Lamparska, Beata Chrapko

**Affiliations:** 1 Department of Pediatric Pulmonology and Rheumatology, Medical University of Lublin, Gębali 6, 20-093 Lublin, Poland; 2 Department of Pediatric Hematology, Oncology and Transplantology, Medical University of Lublin, Lublin, Poland; 3 Laboratory of Genetic Diagnostics, Medical University of Lublin, Lublin, Poland; 4 Department of Pediatric Radiology, Medical University of Lublin, Lublin, Poland; 5 Department of Nuclear Medicine, Medical University of Lublin, Lublin, Poland

**Keywords:** Aortic diseases/diagnostic imaging, Aorta abdominal/abnormalities, Positron emission tomography, Takayasu arteritis, Williams–Beuren syndrome

## Abstract

The differential diagnosis in children with the systemic vasculopathy is still a challenge for clinicians. The progress in vascular imaging and the latest recommendations improve the diagnostic process, but only single reports describe the use of new imaging tests in children. The publication aims to demonstrate the important role of 18F-fluoro-2-deoxy-d-glucose (FDG) positron emission tomography combined with anatomical computed tomography angiography (PET/CTA) imaging in the case of a 15-year-old boy with chest pain, intermittent claudication, hypertension and features of middle aortic syndrome in computed tomography angiography (CTA). The patient was suspected to have Takayasu arteritis, but was finally diagnosed with Williams–Beuren syndrome. The case indicates that the FDG PET/CT imaging might be essential in the diagnostic process of middle aortic syndrome in children. We suggest that this imaging technique should be considered in the diagnostic process of systemic vasculopathy particularly in children.

## Introduction

The middle aortic syndrome is a clinical condition characterized by segmental or diffuse narrowing of an abdominal or distal descending thoracic aorta, with varying degrees of involvement of renal and visceral branches. The syndrome may have different aetiology. The majority of cases are idiopathic, but some are associated with genetic diseases, among them Williams–Beuren syndrome. The condition also develops by acquired inflammatory diseases such as Takayasu arteritis or infection [1]. Regardless of the cause, progressive changes in the vessels may lead to serious complications. The proper and early diagnosis is important for an effective intervention.

Childhood Takayasu arteritis is the most common large vessel vasculitis in children. The diagnosis is difficult since disease rarely affects children and some patients develop uncommon manifestations and associated diseases that may contribute to the delayed diagnosis [2–6].

European League Against Rheumatism (EULAR)/Paediatric Rheumatology International Trials Organisation (PRINTO)/Paediatric Rheumatology European Society (PRES) classification criteria for childhood Takayasu arteritis were published in 2010 [7].

Diagnostic algorithms have been proposed to improve the differentiation causes of vasculopathy [5, 8].

The Williams–Beuren syndrome is a congenital, genetically conditioned multisystemic disorder, caused by a deletion on the long arm of chromosome 7, in the region 7q11.23. Cardiovascular abnormalities occur frequently in consequence of elastin insufficiency. The syndrome is also characterized by dysmorphic features, idiopathic hypercalcemia, or intellectual disability [9–12].

The diagnosis of vasculopathy in the middle aortic syndrome is based on clinical, laboratory and angiographic imaging. Computed tomography angiography (CTA) and magnetic resonance angiography (MRA) can delineate the characteristic changes in vessel anatomy, such as wall thickening, wall enhancement, and alternating focal areas of luminal narrowing and dilatation [8, 13–15]. However, the 18F-fluoro-2-deoxy-d-glucose (FDG) positron emission tomography combined with anatomical CT angiography (PET/CTA) may be the first imaging examination to help identify the occurrence of inflammation in the vascular walls. FDG PET/CTA have the potential to recognize early vessel wall inflammation and may also detect vasculitis before anatomic changes are recognizable at CTA or MRA [8, 13, 14].

EULAR last recommendations from 2018 for imaging in large vessel vasculitis for Takayasu arteritis advise the use of FDG PET/CTA as an alternative imaging modality, particularly in comparison with MRA. FDG PET/CTA might be particularly valuable in the case of patients with unspecific symptoms to detect alternative causes of illness [16].

Also, the recent guidelines in large vessel vasculitis imaging, elaborated by SNMMI (Society of Nuclear Medicine and Molecular Imaging), EANM (European Association of Nuclear Medicine) and PIG (PET Interest Group), pointed to the important value of FDG PET/CTA in the diagnostic process of Takayasu arteritis [17].

The publication aims is to demonstrate the important role of the FDG PET/CTA imaging in the case of a 15-year-old boy with chest pain, intermittent claudication, hypertension and features of middle aortic syndrome in CTA.

## Search strategy

A systematic literature search was performed using electronic literature databases (PubMed/Medline, Scopus database). MESH terms as well as search term combinations were used. Publications published between 2000 and February 2019 in English reporting on paediatric patients suffering from Takayasu arteritis, Williams–Beuren syndrome or middle aortic syndrome that featured the use of PET/CTA in the diagnostic process were included.

Our search results contained a few articles [5, 18–20] (Table 1), and even those were only featuring the use of the FDG PET/CTA method in the diagnosis of childhood Takayasu arteritis—and no articles mentioning this method in the diagnosis of Williams–Beuren syndrome or middle aortic syndrome.

**Table 1:** The previous literature of childhood Takayasu arteritis that featured the use of FGD PET/CTA in the diagnostic process

References	Number of patients	Median age	Diagnosis	Imaging tests	Result of PET
Karapolat [18]	1 (in 21 patients age > 18 years)	18	Takayasu arteritis	FDG PET/CTA	Intense uptake of FDG in the descending aorta, SUV max 4.4
Eleftheriou [5]	4	1.3–17 years	Takayasu arteritis	FDG PET/CTA	Intense uptake, mostly in the ascending aorta, in 2 of 4
Takeishi [19]	1	15	Takayasu arteritis	FDG PET/MRA	Intense uptake of FDG in the aortic root
Fan [20]	7	12–14 years	Takayasu arteritis	FDG PET/CTA	Mean SUV max: 2.8 ± 0.8

*SUV* standardized uptake value, *max* maximum

## Case report

**Fig. 1:**
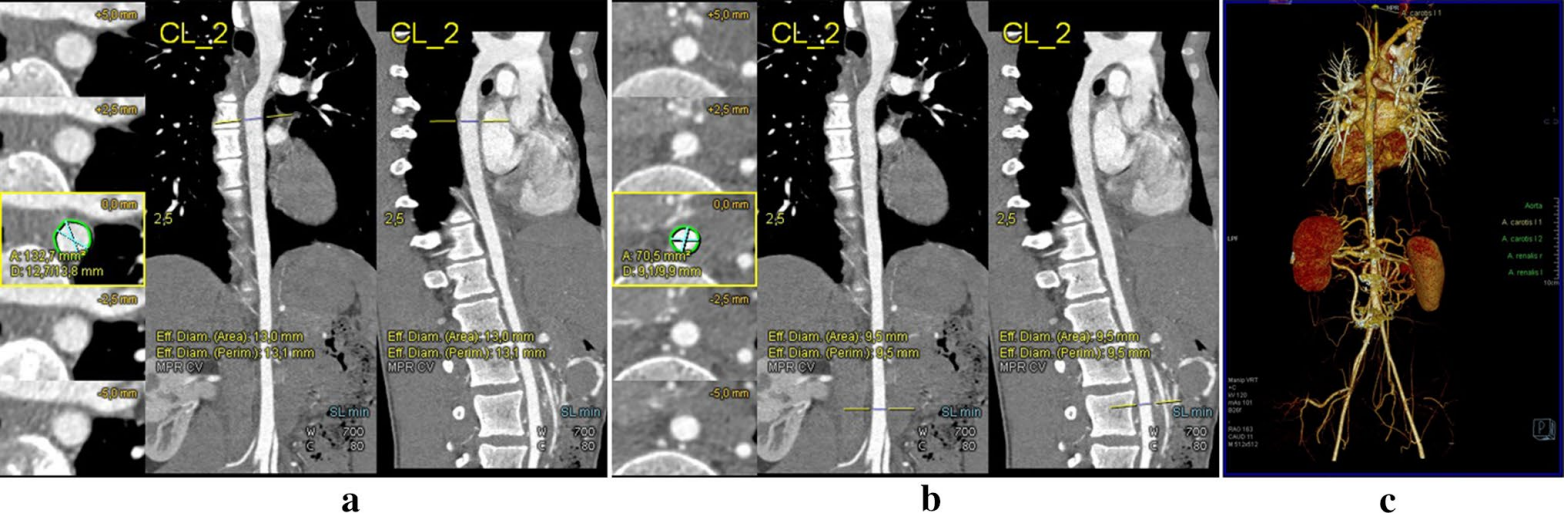
**a, b, c** Computed tomography angiography (CTA): long-segment coarctation of descending aorta beginning just after branching of the left subclavian artery. **a, b** Maximum intensity projection (MIP) reconstruction showing narrowing of the descending aorta at different levels. **c** Volume rendering technique (VRT)

**Fig. 2:**
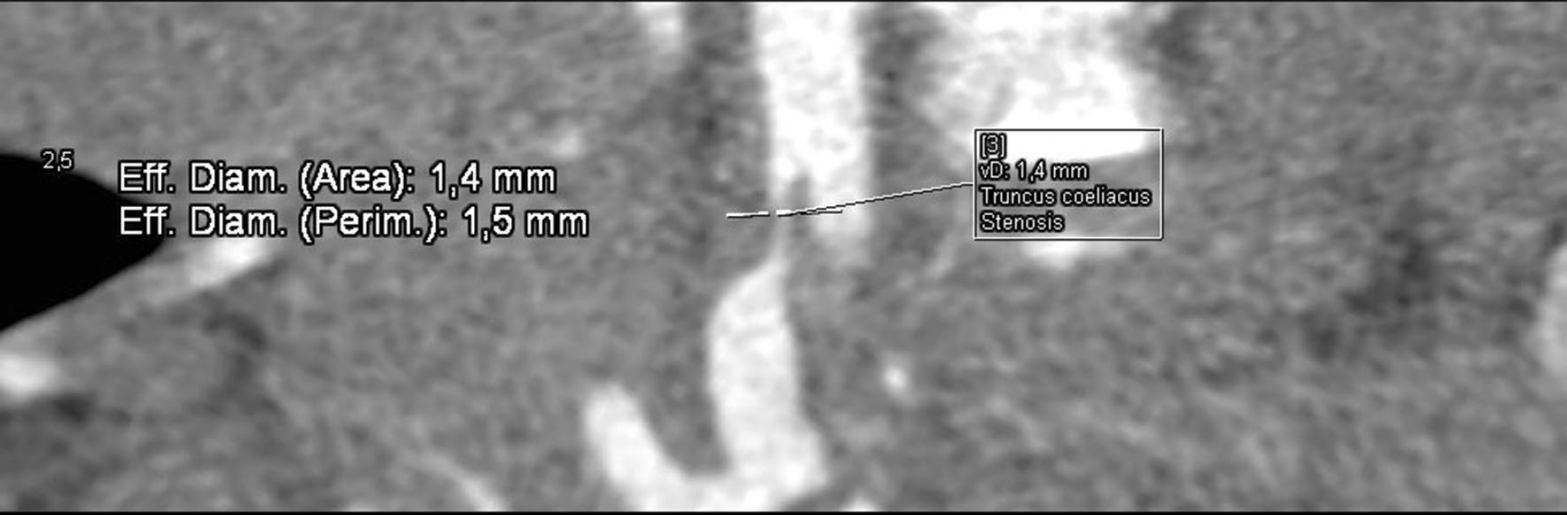
Computed tomography angiography (CTA), maximum intensity projection (MIP) reconstruction. Stenosis of celiac trunk to the diameter of 1.4 mm

The 15-year old male patient was referred to the rheumatology department with suspected Takayasu arteritis. The first symptoms were palpitations and chest pain after physical exertion. They began to occur a few weeks prior to hospitalization. The boy had been diagnosed with a mild intellectual disability in early childhood without suspected genetic disease. Subsequently, the patient was evaluated by a cardiologist and a nephrologist—they diagnosed aortic valve regurgitation, mitral valve insufficiency, and hypertension. CTA with three-dimensional reconstruction and multiplanar presentation (MIP) revealed long segment coarctation of descending aorta beginning just after branching of the left subclavian artery, and involving the origin of its abdominal branches—the celiac trunk, the superior mesenteric artery and both renal arteries, whereas the inferior mesenteric artery was spared. Collateral vessels were also visualized. Carotid and subclavian arteries had no segmental or ostial narrowing; however, carotid arteries appeared rather narrow in comparison with subclavian arteries. There were no signs of vascular wall enhancement or thickening notes in patient’s CTA scans (Figs. 1, 2).

During the hospitalization in the rheumatology ward, the patient was capable of tolerating moderate physical exertion and complained of muscle pain in lower limbs induced by physical activity. Physical examination revealed a deficit of pulse in the peripheral arteries of the hands and some minor dysmorphic facial features. The blood pressure varied from 129/86 mmHg to the maximum 170/110 mmHg and was greater than the 95th centile for the patient height. There was a difference in the blood pressure between the left and right upper limbs of more than 10 mmHg.

Markers of inflammation, erythrocyte sedimentation rate (ESR) and C-reactive protein (CRP), were normal, as well as the results of laboratory tests including morphology, haemostasis, anti-neutrophil cytoplasmic antibodies (ANCAs), antinuclear antibodies (ANA), calcium and potassium concentrations and urine analysis. Ultrasound of the abdominal cavity and chest X-ray showed no abnormalities.

The patient presented characteristic clinical symptoms and met four classification criteria for childhood Takayasu arteritis [7]: angiographic abnormalities, pulse deficit and intermittent claudication, blood pressure discrepancy and hypertension.

A lack of acute phase reactants in blood tests, good general condition and a lack of other systemic vasculitis symptoms were the reasons for the subsequent diagnostic tests. FDG PET/CTA was performed.

**Fig. 3:**
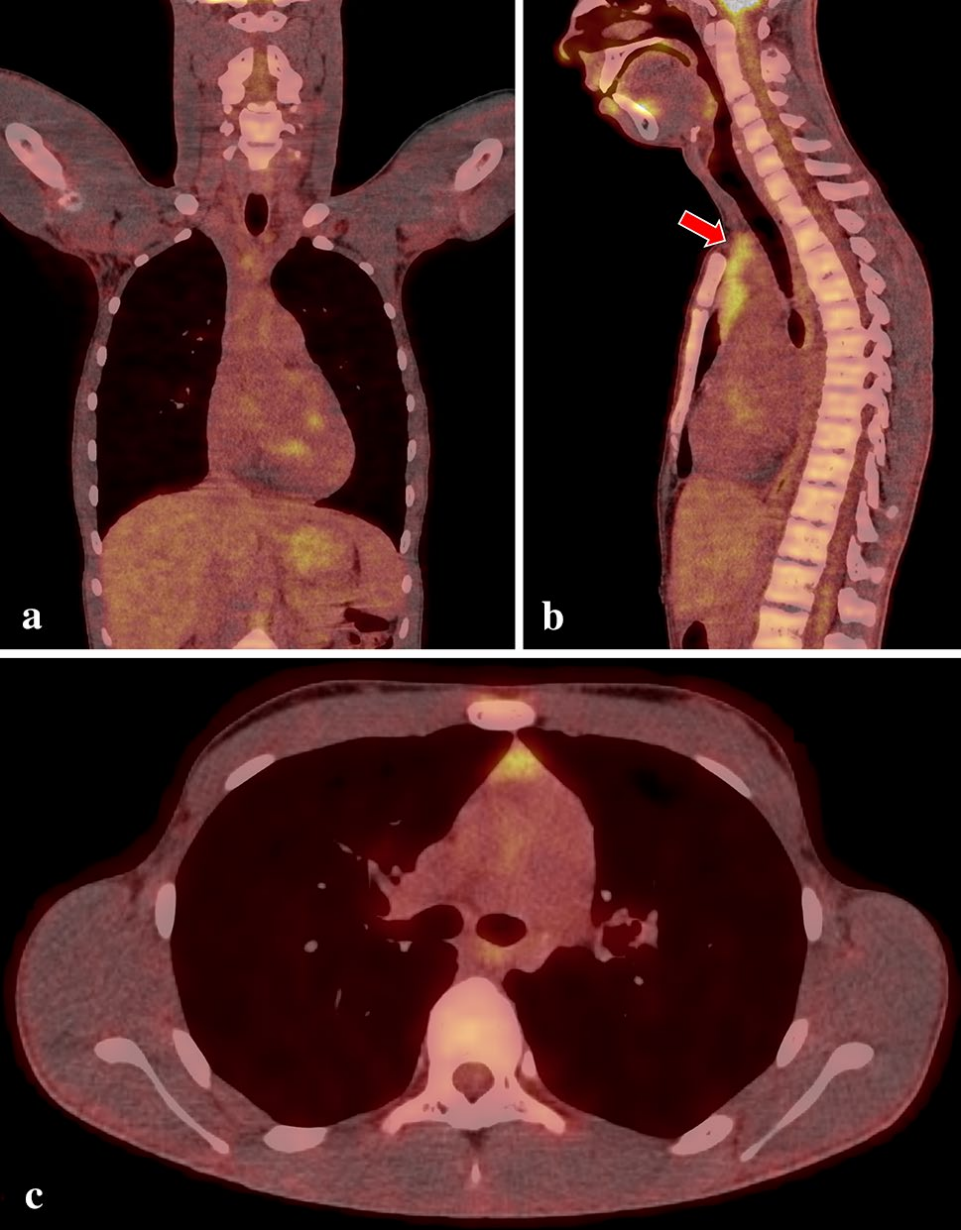
Normal distribution of FDG in PET/CTA in 15-year-old-boy; fused image of the neck and torso in coronal (**a**), sagittal (**b**) and axial (**c**) views. The red arrow on **b** shows normal activity in the thymus in the 15-year-old subject

**Fig. 4:**
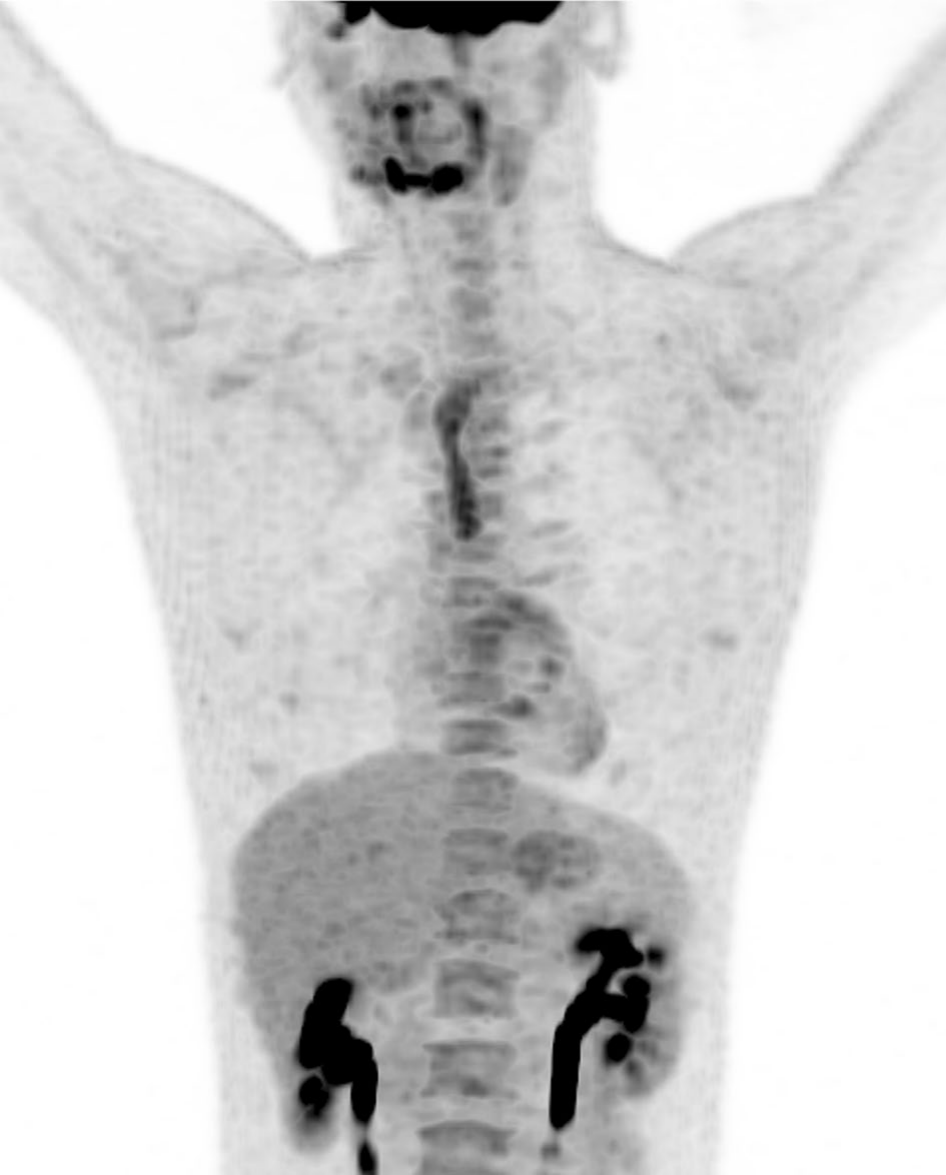
Maximum intensity projection (MIP) of FDG PET/CTA of same patient, physiologic, high activity of FDG in the thymus

Due to the suspicion of the active phase of Takayasu arteritis, high vessel uptake of FDG in PET/CTA was expected, but no glucose metabolic abnormalities were noticed in that study (Figs. 3, 4). In the absence of typical inflammatory patterns in the laboratory tests and FDG PET/CTA, causes other than systemic vasculopathy were considered.

Because of the numerous mild dysmorphic features noticed in the physical examination—narrow palpebral fissures, broad nasal tip, long philtrum, thickened earlobes, webbed neck, drooping shoulders, narrow chest, broad toes, minor increased muscle tone and postural defect—the patient was referred to genetic counselling. The examination of patient revealed mild dysmorphic facial features, but no calcium level disturbances, nor any urinary tract disease. Moreover, no hypermobility of the joints was found.

Based on clinical symptoms and imaging results, the Williams–Beuren syndrome was suspected before performing multiplex ligation-dependent probe amplification (MLPA) test.

MLPA was performed on deoxyribonucleic acid (DNA) from the peripheral blood lymphocytes, extracted with QIAamp DNA Blood Mini Kit (Qiagen, Germany). Salsa Probe mix P029-B1 for Williams–Beuren syndrome (WBS) region (MRC-Holland, Amsterdam, The Netherlands) was used. This kit contains ten probes detecting sequences in the gene coding elastin protein (ELN gene), 15 probes that detect sequences outside ELN gene but within the commonly deleted WBS region, and 5 probes in 7q11.23 but outside the WBS region. Data analysis was made by GeneMarker V2.7.0. The MLPA study performed using a set of probes for region 7q11.23 confirmed the presence of deletions. The results of the molecular tests led to the diagnosis of Williams–Beuren syndrome. Correct MLPA results from both parents confirmed that the deletion in the patient’s 7q11.23 region is de novo*.*

Currently, the patient is under multi-disciplinary care; he remains in the course of therapy with antihypertensive drugs, and did not require cardiac surgery interventions so far.

## Discussion

The symptoms indicating the middle aortic syndrome in children are rare and might be caused by a number of disorders with different aetiologies. Recent metanalysis of 650 cases of children with the middle aortic syndrome demonstrated that most cases are idiopathic (64%), 15% are associated with genetic syndromes like neurofibromatosis type 1 or Williams–Beuren syndrome, and 17% are related to inflammatory diseases, Takayasu arteritis or nonspecific large vessel arteritis [1]. These conditions were considered in the differential diagnosis of the patient (Table 2).

**Table 2:** Clinical, laboratory and imaging features, characteristic of Takayasu arteritis and Williams–Beuren syndrome in the described patient

Symptoms		Takayasu arteritis	Williams–Beuren syndrome
Clinical features	General symptoms	Palpitations and chest pain after physical exertion muscle pain in lower limbs	Mild intellectual disability in early childhood minor dysmorphic facial features
	Symptoms of cardiovascular disease	Hypertension deficit of pulse in the peripheral artery blood pressure discrepancy	Aortic valve regurgitation, mitral valve insufficiency,
Laboratory features	Markers of inflammation		Normal ESR and CRP
Imaging	CTA with MIP	Long-segment coarctation of descending aorta, involving its abdominal branches—the celiac trunk, the superior mesenteric artery and both renal arteries carotid arteries appeared rather narrow in comparison with subclavian arteries	
	FDG PET/CTA		Normal vessel uptake of FDG
Genetic test			MLPA—deletion in 7q11.23

Childhood Takayasu arteritis is the most common large vessel vasculitis in children. This acquired, chronic, idiopathic disease is characterized by intramural granulomatous inflammation of the aorta and its major branches, especially the proximal and occasionally the pulmonary arteries as well. Autoimmune inflammation of the arterial wall and intimal proliferation lead to wall thickening, stenotic or occlusive lesions, and thrombosis. Destruction of the elastic and muscular layers causes aneurysms and dissection. These processes lead to progressive limb or organ dysfunction secondary to ischemia. There is a strong female predominance, with the high prevalence in the 3rd and 4th decades of life, although the beginning of the disease may develop earlier, including in the childhood years [2–6]. The diagnosis is based on clinical signs and typical angiographic abnormalities in the aorta and its major branches after excluding other causes.

The acute phase of the disease is characterized by non-specific symptoms—such as hypertension, headache, fever, muscle pain, arthralgia, night sweats and weight loss. Due to the non-specific symptoms and the absence of specific laboratory parameters, the disease is often unrecognized in this phase. If untreated, during the next phase, the disease affects the aorta and its main branches. Laboratory investigations particularly high values of inflammation markers should support the diagnosis. Characteristic features of the late, occlusive phase are limb ischemia or absent pulses, vascular bruits, hypertension as a consequence of renal artery stenosis or aortic narrowing and aortic fibrosis, mesenteric angina, retinopathy, aortic regurgitation and neurologic symptoms secondary to hypertension or ischemia. An aortic valve insufficiency and congestive heart failure has been reported in a significant proportion of patients [6, 21–28]

The current EULAR/PRINTO/PRES validated classification criteria for childhood Takayasu arteritis are characterized by high sensitivity and specificity assessed as 100% and 99.9%, respectively [7]. The mandatory criterion is the typical angiographic abnormality of the aorta or its main branches and pulmonary arteries found in angiography (conventional, CTA or MRA): aneurysm/dilatation, narrowing, occlusion or thickened arterial wall not due to fibromuscular dysplasia, or similar causes, usually focal or segmental.

An angiographic classification system on the basis of the location of the lesions exists: type I (branches of the aortic arch, classically associated with the typical pulseless disease), type IIa (ascending aorta, aortic arch and its branches), type IIb (ascending aorta, aortic arch and its branches, and thoracic descending aorta), type III (thoracic descending aorta, abdominal aorta, and/or renal arteries), type IV (abdominal aorta and/or renal arteries) and type V (combined features of types IIb and IV) [29].

According to the classification criteria, presence of one of the following symptoms is required for diagnosis: pulse deficit or claudication, blood pressure discrepancy in any limb, bruits, hypertension or elevation of an acute phase reactants. These symptoms are highly specific (74.7%, 63.5%, 63.2%, 95%) and sensitive (99.1%, 99.6%, 90.5%, 14.1%, respectively) for the diagnosis of Takayasu arteritis [7].

A characteristic laboratory feature for childhood Takayasu arteritis that did not occur in our patient is elevated acute phase reactants: ESR or CRP [7]. In consensus evaluations, abnormal acute phase reactants were observed at the time of diagnosis in 87% of patients [7].

In the described case, a lack of acute phase reactants, general good condition and a lack of other systemic symptoms like a fever and weight were indicators for extending diagnostic tests.

The importance of FDG PET/CTA for the diagnosis of Takayasu arteritis has been proven in several newly published studies [30–34], but rare for childhood-onset disease [5, 18–20] (Table 1). FDG PET/CTA might be particularly valuable in patients with unspecific symptoms to detect alternative causes of systemic vasculopathy [16].

One recent meta-analysis [13], the aim of which was to evaluate the effectiveness of imaging modalities for the management of Takayasu arteritis, showed the sensitivity and specificity of FDG PET/CTA for disease activity at 81% and 74%, respectively. However, among the ten studies selected for the analysis, there was none carried out in the paediatric population.

The cellular mechanisms of FDG uptake follow those of native glucose and depend on the expression of membrane glucose transporter proteins (GLUT). After FDG internalization, it is phosphorylated to FDG-6-phosphate, but in contrast to native glucose, FDG-6-phosphate does not enter the glycolytic pathway and is trapped within the cell in the unchanged form. Both glucose and its structural analogue FDG are also strongly absorbed by activated leucocytes. The accumulation of FDG in activated leucocytes enables the imaging of infections and inflammations. Therefore, FDG PET/CTA is designated for diagnosis of fever of unknown origin (FUO), inflammatory bowel disease, osteomyelitis, spondylodiscitis, vasculitis, vascular graft infections and assessment of inflamed and vulnerable plaque [17].

In the described patient, the presence of an active inflammatory process was not confirmed nor in laboratory tests as increased ESR or CRP, nor as increased vessels uptake of FDG in the FDG PET/CTA. The differentiation was continued to establish noninflammatory cause of vasculopathy.

The Williams–Beuren syndrome is a congenital, genetically conditioned multisystemic disorder, caused by a deletion in the long arm of chromosome 7, in the region 7q11.23. The syndrome occurs at a frequency of 1 in 10 000 live-borns. The main symptom at diagnosis is stenosis of the aortic or other vessels. Cardiovascular abnormalities occur frequently, approximately in 90% of patients. Arterial abnormalities include localized or diffuse narrowing of elastic arteries. Supravalvular aortic stenosis and supravalvular pulmonary stenosis are the prevalent cardiovascular anomaly. Other less common abnormalities include stenosis of the abdominal and thoracic aorta and mid-size arteries, such as the renal arteries and the coronary arteries. Imaging examinations demonstrated generalized arterial wall thickening present even in nonstenotic areas of the arterial tree [1, 12, 35–37].

The deletion of the ELN gene is presumed to be the main cause of arterial stenosis. Both human and animal studies suggest that elastin is required for the terminal differentiation and quiescence of vascular smooth muscle cells. Elastin is thought to be necessary for terminal differentiation and inactivity of vascular smooth muscle cells [38].

Cardiovascular CTA is considered the best imaging method and well validated for visualization and characterization of the coronary and extracardiac vasculature in the Williams–Beuren syndrome [35].

To our knowledge, this is the first description of the FDG PET/CTA imaging of peripheral vessels in the Williams–Beuren syndrome with normal uptake of FDG.

The Williams–Beuren syndrome is also characterized by dysmorphic features, idiopathic hypercalcemia, or intellectual disability. The unique phenotypic features are described as elfin face, with broad forehead, a short nose with a broad nasal tip, full cheeks, and wide mouth with full lips. With progressing age, the proportions of the face change to appear longer and more gaunt [1, 9, 39, 40]. Most of the adults displayed premature greying of the hair. This feature, together with an earlier-than-expected onset of cataracts and high-frequency sensorineural hearing loss, suggested mild accelerated ageing, which may additionally complicate the long-term course of the syndrome in older adults [40, 41].

The next common problem in Williams–Beuren syndrome is hypercalcemia often associated with hypercalciuria, arterial calcification, and nephrocalcinosis. In 20–35% of patients, structural defects of the genitourinary system, such as ectopic kidney, horseshoe kidney, and bladder diverticula, occur [42]. Hypermobility of the joints often delays motor development, causing hyper-reflexed thighs, bent knees, and spinal kyphosis [40].

The described patient had expressed mild dysmorphic facial features, but no calcium level disturbances, nor any urinary tract disease, nor any hyperflexibility of the joints was found.

The deletions in the region of 7q11.23 evoke a very specific behavioural profile which consists of the moderate intellectual disability which is characterized by serious difficulties in processing visuospatial tasks, relatively strong language skills and a hypersocial personality, sensitivity to sound, and fondness of music. Studies of the intelligence quotient (IQ) in people with Williams–Beuren syndrome show a range of IQ scores from 40 to 100, with average scores of 50–60. Psychiatric and psychological problems are common, including attention deficit hyperactivity disorder (ADHD) and anxiety disorders, especially specific phobias [43]. The described boy had minor intellectual challenges diagnosed from early childhood. Other disorders have not been confirmed.

Understanding Williams–Beuren syndrome and knowing its clinical features are prerequisites to establishing a correct diagnosis; hence, a team of different specialists must be involved in the diagnostic procedure. Any suspicion of a genetic condition should be verified in a consultation with an experienced clinical geneticist.

## Conclusions

In each case of middle aortic syndrome, different aetiology should be thoroughly considered. We demonstrate the importance of the FDG PET/CTA imaging in the case of a 15-year-old boy with suspected childhood Takayasu arteritis and final diagnosis of Williams–Beuren syndrome.

Multidisciplinary cooperation is a prerequisite for making the correct diagnosis. Any suspicion of a genetic condition should be verified by consultation with an experienced clinical geneticist.

The case indicates that the FDG PET/CTA imaging might be essential in the diagnostic process of middle aortic syndrome in children. We suggest that this imaging technique should be considered in the diagnostic process of systemic vasculopathy particularly in children.
